# Radiomic prediction of radiation pneumonitis on pretreatment planning computed tomography images prior to lung cancer stereotactic body radiation therapy

**DOI:** 10.1038/s41598-020-77552-7

**Published:** 2020-11-24

**Authors:** Taka-aki Hirose, Hidetaka Arimura, Kenta Ninomiya, Tadamasa Yoshitake, Jun-ichi Fukunaga, Yoshiyuki Shioyama

**Affiliations:** 1 Division of Radiology, Department of Medical Technology, Kyushu University Hospital, 3-1-1, Maidashi, Higashi-ku, Fukuoka, 812-8582 Japan; 2 Faculty of Medical Sciences, Kyushu University, 3-1-1, Maidashi, Higashi-ku, Fukuoka, 812-8582 Japan; 3 Department of Health Sciences, Graduate School of Medical Sciences, Kyushu University, 3-1-1, Maidashi, Higashi-ku, Fukuoka, 812-8582 Japan; 4 Department of Clinical Radiology, Graduate School of Medical Sciences, Kyushu University, 3-1-1, Maidashi, Higashi-ku, Fukuoka, 812-8582 Japan

**Keywords:** Cancer, Lung cancer, Non-small-cell lung cancer, Oncology

## Abstract

This study developed a radiomics-based predictive model for radiation-induced pneumonitis (RP) after lung cancer stereotactic body radiation therapy (SBRT) on pretreatment planning computed tomography (CT) images. For the RP prediction models, 275 non-small-cell lung cancer patients consisted of 245 training (22 with grade ≥ 2 RP) and 30 test cases (8 with grade ≥ 2 RP) were selected. A total of 486 radiomic features were calculated to quantify the RP texture patterns reflecting radiation-induced tissue reaction within lung volumes irradiated with more than x Gy, which were defined as LVx. Ten subsets consisting of all 22 RP cases and 22 or 23 randomly selected non-RP cases were created from the imbalanced dataset of 245 training patients. For each subset, signatures were constructed, and predictive models were built using the least absolute shrinkage and selection operator logistic regression. An ensemble averaging model was built by averaging the RP probabilities of the 10 models. The best model areas under the receiver operating characteristic curves (AUCs) calculated on the training and test cohort for LV5 were 0.871 and 0.756, respectively. The radiomic features calculated on pretreatment planning CT images could be predictive imaging biomarkers for RP after lung cancer SBRT.

## Introduction

Stereotactic body radiotherapy (SBRT) is commonly administered for early stage non-small-cell lung cancer (NSCLC) to reduce treatment volumes and facilitate hypofractionation with delivery of large daily tumor doses^1,2^. The reported survival rate of SBRT is comparable to that of surgery^2^. However, radiation-induced pneumonitis (RP) is the most frequent acute pulmonary toxicity following SBRT for lung cancer. Although most patients develop asymptomatic grade 1 pneumonitis, clinically symptomatic pneumonitis is often observed^3–5^. Thus, previous studies have used clinical and dosimetric data to attempt to predict RP risk after radiation therapy (RT) for lung cancer^6–11^. Biological markers such as serum Krebs von den Lungen-6 (KL-6) and surfactant proteins-D (SP-D) levels are reportedly useful for the prediction of RP after SBRT treatment^6,7^. Other studies have concluded that RP incidence and grade are significantly related to various Vx (percentage lung volume receiving > x Gy) such as V20 and mean lung dose (MLD)^8–11^. Thus, the present study considered the dosimetric effects on RP development based on regions of interest (ROIs) segmented by dosimetric information.

Recent studies have reported improved RP prediction by applying machine learning. Cunliffe et al.^12^ investigated the relationship between radiation dose and changes in lung radiomic features on pre- and post-treatment computed tomography (CT) images in patients who received curative radiation doses for esophageal cancer, using multiple features in a classifier. Moran et al. reported that changes in radiomic features on pre- and post-SBRT CT images were significantly correlated with radiation oncologist-scored post-SBRT lung injury^13^. Cui et al.^14^ proposed a combination of handcrafted features with latent variables selected from 230 variables, including clinical factors and biomarkers in machine learning. However, to our knowledge, no studies have predicted the RP risk after lung cancer SBRT from the radiomic features obtained only from pretreatment planning CT images. Achieving the RP prediction with only pretreatment planning CT images prior to radiation delivery may be useful for selecting treatment options and creating treatment plans.

Previous studies have demonstrated the ability of radiomics to provide a quantitative evaluation of lung tissue reaction to radiation dose on pre- and post-RT CT images and assess the occurrence of RP^12,13^. We hypothesized that the radiomic features calculated from only pretreatment planning CT images could quantify RP texture patterns reflecting radiation-induced tissue reaction, thereby predicting RP. Therefore, the purpose of this study was to develop a predictive model for RP after lung cancer SBRT using radiomic features for lung ROI segmented by dosimetric information on pretreatment planning CT images.

## Materials and methods

### Patient data

This retrospective study was performed with the ethical approval of the institutional review board of our hospital. 245 training cases and 30 test cases (a total of 275 patients) were selected as a training and test cohorts, respectively, from two different terms and planning CT scanners. The training cohort included 245 patients who underwent SBRT for NSCLC between August 2003 and July 2013 (median dose: 48 Gy; median age: 77 years; range: 52–91 years; TNM classification: T1–2, N0, M0). Twenty-two of these patients had grade 2 or higher RP. The test cohort consisted of 30 patients including eight RP cases and 22 non-RP cases who underwent SBRT for NSCLC between April 2014 and March 2018 (median dose: 48 Gy; median age: 74 years; range: 54–90 years; TNM classification: T1–2, N0, M0). The patients’ characteristics are summarized in Table 1. The patients of the training cohort were scanned using a planning CT (Mx 8000, Philips Healthcare, Amsterdam, The Netherlands) with a tube voltage of 120 kV, an in-plane pixel size of 0.98 mm, and a slice thickness of 2.0 mm for treatment planning. The patients of the test cohort were acquired on a different CT scanner for the training cohort (Aquilion Prime, Canon Medical Systems, Otawara, Japan) for validation under the same conditions as the training cohort. Three-dimensional conformal radiation therapy (3DCRT) plans with non-coplanar fields were created using a commercially available radiation treatment planning (RTP) system (Eclipse; Varian Medical Systems Inc., Palo Alto, USA). RP grades were scored using the Common Terminology Criteria for Adverse Events version 4.0 (CTCAE v.4.0) based on clinical assessment and imaging^15^.

**Table 1:** Patient characteristics.

Characteristics	Training cohort (n = 245)	Test cohort (n = 30)
No. of patients	245	30
**RP grade**		
0–1	223	22
≥ 2	22	8
**Gender**		
Male	153	21
Female	92	9
**Age (year)**		
Median	77	74
Range	51–92	54–90
**Tumor stage**		
T1	187	27
T2	58	3
**Tumor diameter (mm)**		
Median	22.8	22.3
Range	10.0–53.0	8.7–42.2
**Radiation dose (Gy)**		
Median (range)	48 (48–60)	48 (48–60)
**Dose and fraction**		
12 Gy × 4 Fr. (48 Gy)	239	25
13 Gy × 4 Fr. (52 Gy)	2	3
6 Gy × 10 Fr. (60 Gy)	4	2
**Dose prescription method**		
Isocenter	144	10
D95 of PTV	101	20

### Overall scheme

**Figure 1:**
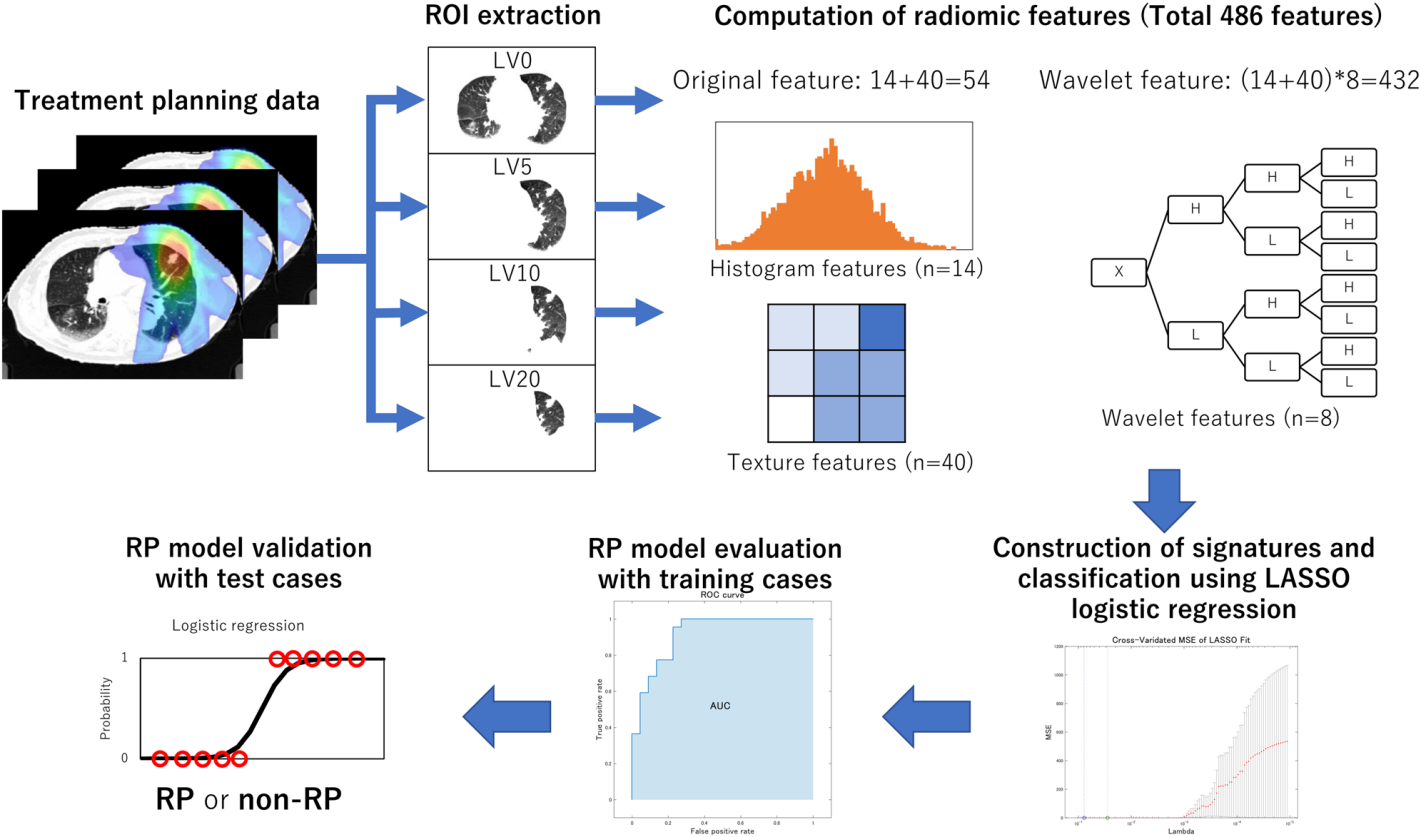
An overall workflow of the proposed scheme for RP prediction.

Figure 1 illustrates the overall workflow of the proposed scheme for RP prediction. First, the ROIs for calculating radiomic features were extracted from the treatment planning data. Four ROIs were created by extracting lung volumes excluding gross tumor volume (GTV) irradiated with more than 0, 5, 10, and 20 Gy for each patient, which were defined as LV0, LV5, LV10, and LV20, respectively. Second, a total of 486 radiomic features, which consisted of 54 original radiomic features (14 histogram-based and 40 texture features) and 432 wavelet-based radiomic features with 8 wavelet decompositions (54 features × 8 wavelet decomposition filters), were calculated from each ROI. Third, the significant features were selected as a signature (set of selected significant features) for each ROI, and then RP predictive models with signatures for each ROI were built to classify patients with and without grade ≥ 2 RP using a least absolute shrinkage and selection operator (LASSO) logistic regression. Finally, the constructed models for each ROI were evaluated with training and test cases by areas under the receiver operating characteristic (ROC) curve (AUC), sensitivity, specificity, and accuracy.

### ROI extraction

The ROIs for calculating radiomic features were extracted using structure data for total lung volumes excluding the GTV and dosimetric data obtained from the treatment planning data. Four ROIs were created by extracting lung volumes irradiated with more than 0, 5, 10, and 20 Gy for each patient, which were defined as LV0, LV5, LV10, and LV20. The image processing was performed using in-house software with MATLAB 2019a (MathWorks).

### Computation of radiomic features

**Figure 2:**
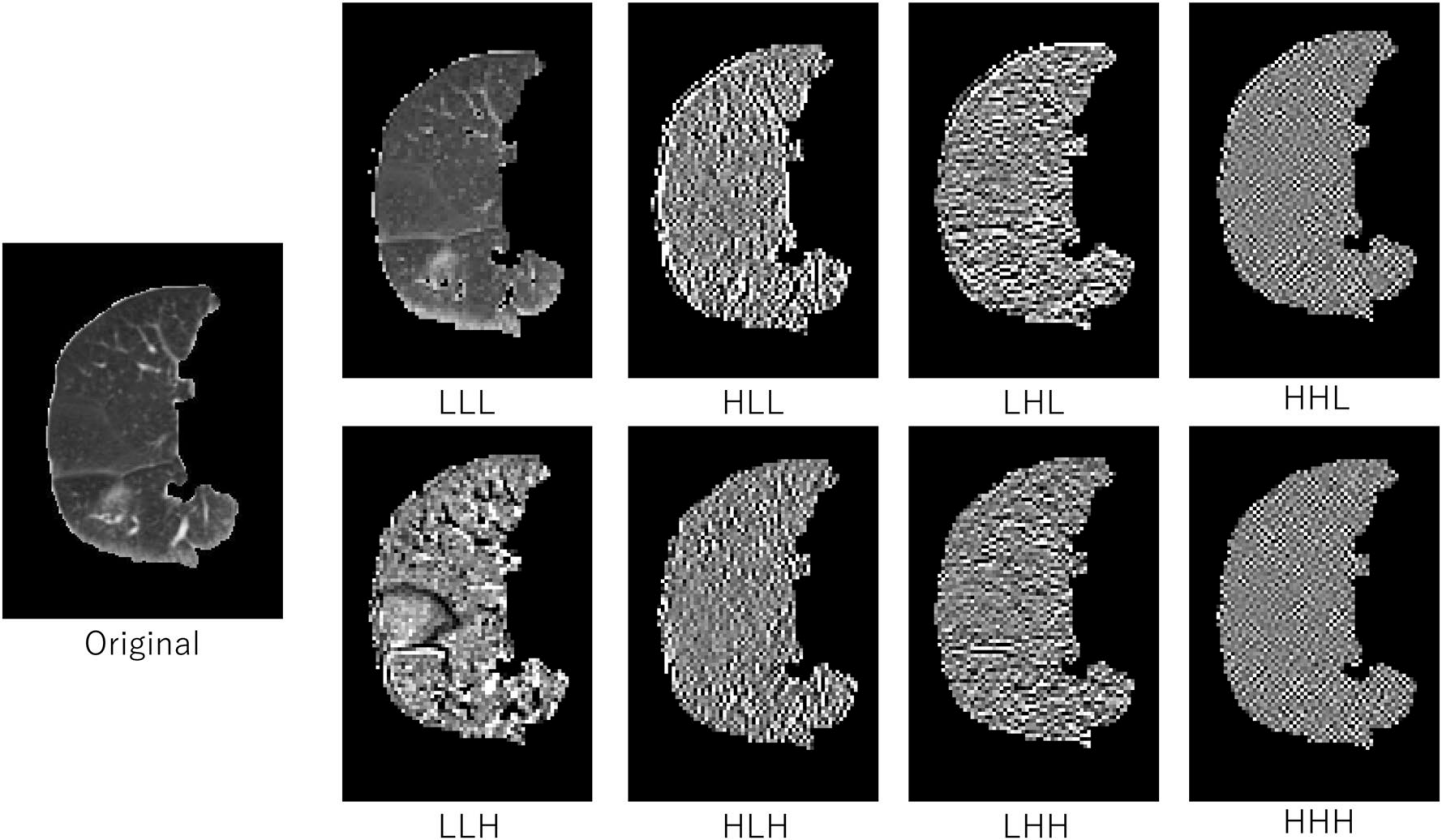
Eight wavelet decomposition images of an original lung volume image by applying either a low-pass filter (scaling function, L) or a high-pass filter (wavelet function, H) in x, y, or z direction, and its corresponding filter.

A total of 486 radiomic features including 54 original features and 432 wavelet-based features were calculated from each ROI in pretreatment planning CT images for quantifying RP texture patterns using the MATLAB-based Radiomics tools package (implemented in MATLAB 2019a, MathWorks)^16,17^. The 54 original radiomic features consisted of 14 histogram-based and 40 texture features. The texture features were calculated from four texture-characterization matrices (i.e., a gray level co-occurrence matrix [GLCM]^18^, gray level run-length matrix [GLRLM]^19^, gray level size-zone matrix [GLSZM]^20^, and neighborhood gray-tone difference matrix [NGTDM]^21^). The 54 original radiomic features are listed in the Supplemental Information (Table S1). Then, 432 wavelet-based radiomic features were derived from the same 54 features as the original features on each of the eight wavelet decomposition images^22^. The wavelet transform can decompose multiscale local lung texture patterns related to RP and non-RP in an image into several low- and high-frequency components^23^. The decomposition was performed by applying either a low-pass filter (scaling function, L) or a high-pass filter (wavelet function, H) in the x, y, or z direction. The eight wavelet decomposition filters consisted of a combination of three using either a low-pass filter (L) or a high-pass filter (H) in each direction. Figure 2 shows the CT images with RP decomposed based on the wavelet analysis. The original image shows the texture patterns different from 7 (HLL to HHH) wavelet decomposition images, although the LLL image is similar to the original image. In this study, we assumed that radiomic features on original images could represent lung texture properties different from wavelet-based radiomic features provided by the wavelet decomposition images.

### Construction of signatures and building RP predictive model using LASSO logistic regression

Although we assumed that original image features could represent lung texture properties different from wavelet-based image features, some features could be linearly dependent, especially features from original and LLL images. However, according to the LASSO theory^24^, that linear dependence does not imply dispensability, and individual dispensability does not imply pairwise dispensability, we decided to employ the original CT images. Nevertheless, to avoid the risk of overfitting on the RP prediction model, the significant features among 486 radiomic features were reduced to a number of features using a LASSO logistic regression with MATLAB 2019a (MathWorks)^25^. This process was repeated 1000 times for each ROI. The radiomic features with the highest frequency were extracted from the 486 radiomic features to build the RP predictive model for each ROI^26^. The RP grades were annotated by 1 for RP = 2 or above and 0 for otherwise, as the teacher data to be inputted into the logistic regression models. The logistic regression models were constructed with the radiomic signatures for each ROI to classify patients with and without grade ≥ 2 RP.

### RP predictive model with dose-volume histogram parameters

A logistic regression model with four dose-volume histogram (DVH) parameters of the lung volumes receiving more than 5, 10, and 20 Gy (V5, V10, V20) and MLD was also constructed for comparisons between DVH and the radiomics models. The most frequently selected significant DVH parameter combination using a LASSO logistic regression, which was similar to the signature construction mentioned above, was used for the RP predictive model.

### Construction of an ensemble averaging model with imbalanced datasets adjustment strategy

**Figure 3:**
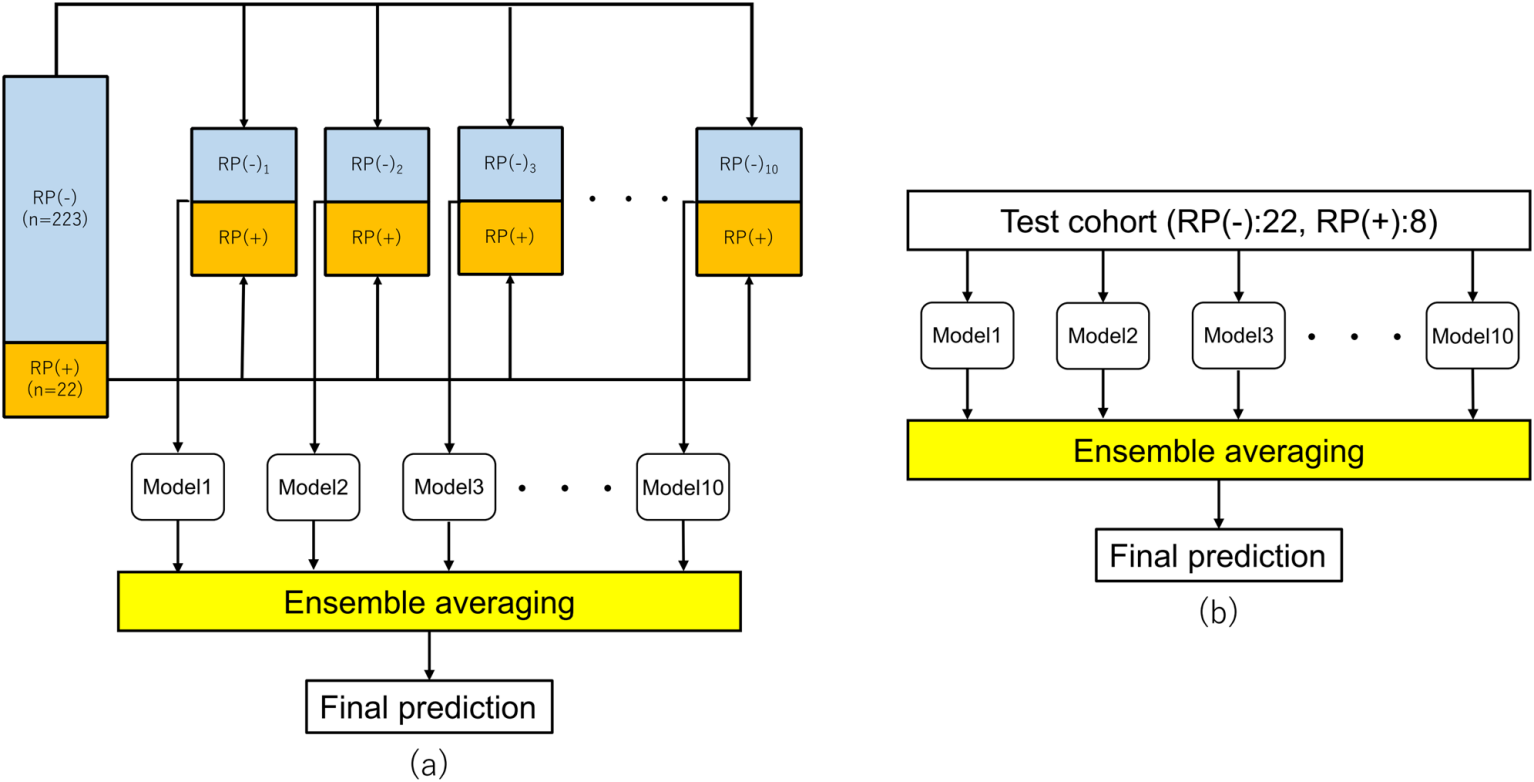
The concept of construction of an ensemble averaging model an imbalance adjustment strategy: (**a**) construction of an ensemble averaging model based on 10 subsets using a training cohort with 245 patients and (**b**) test of an ensemble averaging model using a test cohort with 30 patients.

In this study, only 22 (9%) of the 245 training patients had grade ≥ 2 RP. Imbalanced datasets cause performance loss in the classification model^27^. To address the issue of imbalanced data, this study used an imbalance adjustment strategy adapted from that described by Schiller et al.^28^. As shown in Fig. 3a, the data were partitioned into a collection of balanced subsets. Thus, 10 subsets consisting of all 22 RP cases and 22 or 23 randomly extracted non-RP cases were created from the imbalanced training dataset of 245 patients. The recommended number of features should be generally smaller than around one-tenth of the number of training cases to avoid the overfitting problem^29,30^. Additionally, in leave-one-out cross-validation performed beforehand for each subset with an increasing number of top features for RP prediction, the predictive models with the top four features showed the highest performance (Fig. S1). Therefore, for each subset with each ROI, the top four significant features were selected for the construction of signatures, and 10 predictive models were built with the signatures using LASSO logistic regression. Significant DVH parameter combination was also selected for each subset using LASSO logistic regression for the RP predictive model. Finally, as shown in Fig. 3a, an ensemble averaging model was newly built by averaging the RP probabilities of the 10 predictive models constructed from 10 different subsets made from 245 training cases for each ROI.

### RP predictive model training and testing

The ensemble averaging model for the RP prediction was considered to be trained with 245 training cases. Four significant features were selected in each predictive model for each ROI using LASSO logistic regression. As shown in Fig. 3b, the built ensemble averaging model was tested with 30 test cases in the same manner as the model training. The RP predictive model was evaluated according to the AUC, sensitivity, specificity, and accuracy. The sensitivity, specificity, and accuracy are given by

1 $$ Sensitivity = \frac{TP}{{TP + FN}}{ },{ } $$

2 $$ Specificity = \frac{TN}{{TN + FP}}{ }, $$

and

3 $$ Accuracy = \frac{TP + TN}{{TP + FN + TN + FP}}{ }, $$

where TP, FP, TN, and FN are the numbers of true positives, false positives, true negatives, and false negatives, respectively. The AUC was obtained from the area under the ROC curve, which was a plot of sensitivity against (1-specificity) by changing the discrimination threshold of a classifier system.

### Ethical statement

This retrospective study was performed with the ethical approval of the institutional review board of our hospital. Written informed consent was obtained from all subjects within the dataset collected in our hospital. All of the methods were carried out in accordance with the Declaration of Helsinki.

## Results

Table 2 shows AUCs, sensitivity, specificity, and accuracy of the ensemble averaging model for 245 training and 30 test cases. The AUCs of the RP predictive model for the training cohort with DVH parameters and radiomic signatures for LV0, LV5, LV10, and LV20 were 0.703, 0.868, 0.871, 0.905, and 0.890, respectively. The AUCs for the test cohort were 0.290, 0.557, 0.756, 0.602, and 0.608, respectively. All radiomic models showed higher performance than DVH model.

**Table 2:** AUCs, sensitivity, specificity, and accuracy of the ensemble averaging model for training and test cohorts.

	DVH	LV0	LV5	LV10	LV20
**Training cohort (n = 245)**					
AUC	0.703	0.868	0.871	0.905	0.890
Sensitivity	0.636	0.818	0.818	0.909	0.909
Specificity	0.646	0.691	0.722	0.709	0.731
Accuracy	0.645	0.702	0.731	0.727	0.747
**Test cohort (n = 30)**					
AUC	0.290	0.557	0.756	0.602	0.608
Sensitivity	0.125	0.500	0.500	0.250	0.125
Specificity	0.500	0.636	0.818	0.682	0.909
Accuracy	0.400	0.600	0.733	0.567	0.700

Table 3 shows the top four radiomic features selected most frequently for 10 subsets for each ROI. The radiomic feature of “correlation” computed with GLCM on the original images was selected as the signature for each ROI.

**Table 3:** Top 4 radiomic features selected most frequently for 10 subsets for each ROI.

ROI	#1 RF	#2 RF	#3 RF	#4 RF
LV0	Mean (HLH)	RLV (LHL)	Uniformity (original)	Correlation (original)
LV5	RLV (LHL)	Correlation (original)	RLV (LLH)	Entropy_GLCM (original)
LV10	Correlation (original)	SZLGE (LLL)	Entropy (LLL)	LZHGE (original)
LV20	Skewness (HLL)	LZHGE (original)	Entropy (LLL)	Correlation (original)

RLV, run-length variance; GLCM, gray level co-occurrence matrix; SZLGE, small zone low gray-level emphasis; LZHGE, large zone high gray-level emphasis.

## Discussion

Using the radiomic features for lung ROIs dosimetrically segmented from the pretreatment planning CT images of 275 NSCLC patients, we found that the radiomic predictive models to classify patients with and without grade ≥ 2 RP performed well. In the training cohort, the AUC for the ensemble averaging model with LV10 signatures using the top four radiomic features reached the maximum value of 0.905. In the test cohort, the radiomic predictive model for LV5 reached the highest AUC of 0.756. This model for LV5 also showed a high AUC of 0.871 in the training cohort. Based on these results, the radiomic predictive model for LV5 was considered the best model.

The prediction results of the test cohort were lower than those of the training cohort. In particular, in terms of low sensitivity, the number of RP cases in the training and test cohorts might be insufficient. The difference between CT equipment and breathing methods during image acquisition may also have affected the radiomic features. The CT scans were performed on free breathing in the training cohort, while breath-hold techniques were used in the test cohort.

**Figure 4:**
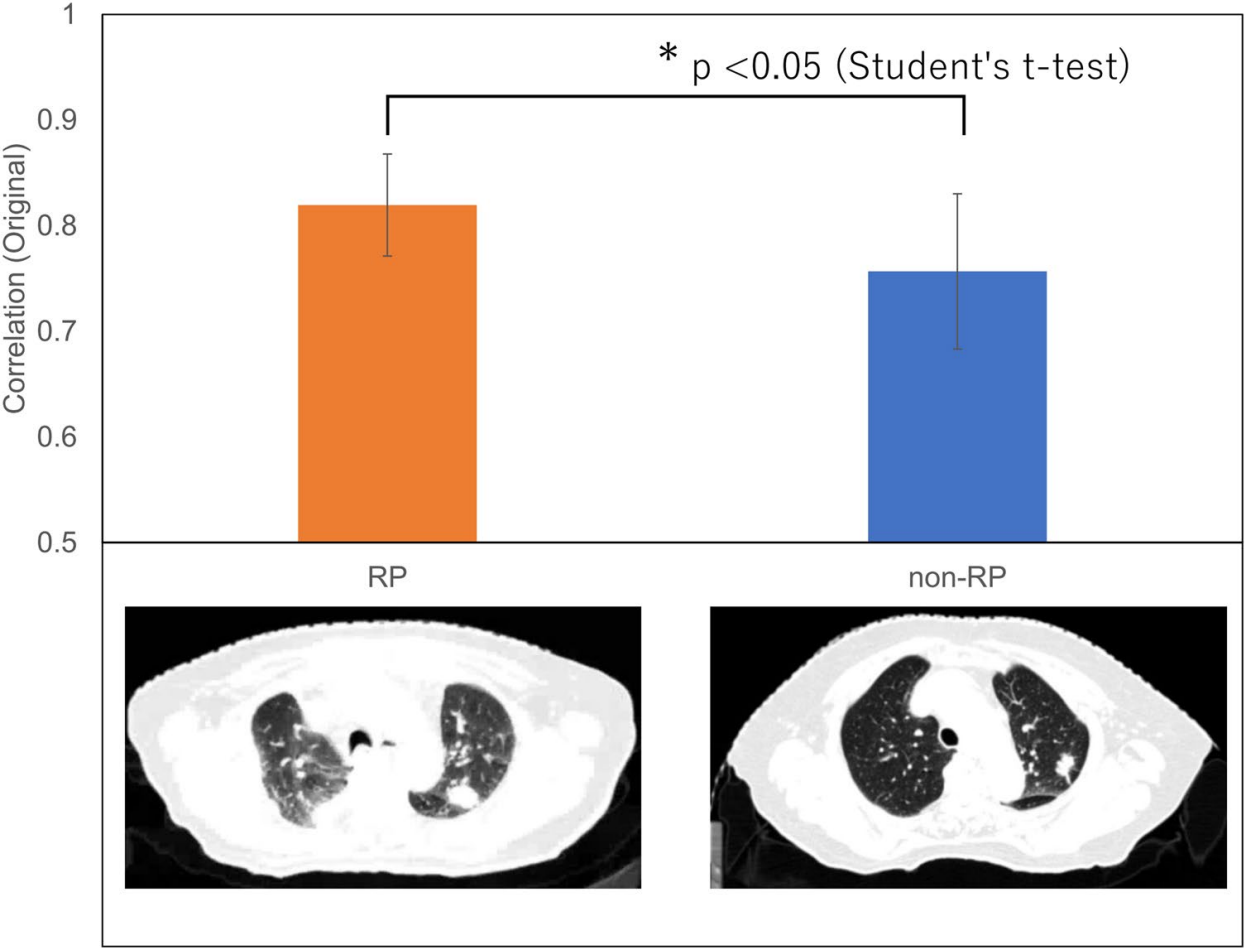
Bar graph of “correlation” values on the original images for LV5 of RP and non-RP cases in the training cohort and an example of pretreatment planning CT images of RP and non-RP cases.

The “correlation” computed with GLCM on the original images was selected as one of the frequently selected features for each ROI in Table 3. Correlation is a measure of how correlated a pixel is to its neighbor over the whole image. Figure 4 shows a bar graph of “correlation” values on the original images for LV5 of RP and non-RP cases in the training cohort and an example of pretreatment planning CT images of RP and non-RP cases. The values of “correlation” of RP cases were significantly higher than those of non-RP cases. These results indicate that the “correlation” on the original images could quantify the RP characteristics different from the one on the wavelet decomposition images and might be one of the imaging biomarkers for RP after lung cancer SBRT.

Previous studies on RP prediction are summarized in Table 4. The previous studies often used DVH parameters such as V20 and MLD as risk factors for RP prediction^8–11,14,31^. Various clinical factors and biomarkers such as cytokines, single nucleotide polymorphisms (SNPs), and microRNA have also been used for RP prediction^14,31^. In the field of radiomics, Cunliffe et al. proposed that dose-dependent texture changes between pre- and post-RT CT images could classify patients with and without grade ≥ 2 RP. When multiple features were combined in a classifier, AUC increased significantly (from 0.59 to 0.84)^12^. Moran et al.^13^ found that changes in radiomic features calculated from follow-up CT images after SBRT for 14 patients were significantly correlated with post-SBRT lung injury scores provided by a radiation oncologist, and that the AUCs using GLCM texture features ranged from 0.689 to 0.750.

**Table 4:** AUCs for different studies using different RP prediction strategies.

Reference	Features (n)	Classification	Methods	AUC	Patient information
Current study	Radiomic features (486)	RP grade ≥ 2	Logistic regression	0.871 (training) 0.756 (test)	SBRT For 275 stage I NSCLC patients
Cunliffe et al.^12^	Radiomic features (20)	RP grade ≥ 2	Logistic regression	0.84	CFRT for 106 stage I–IV esophageal cancer patients
Moran et al. ^13^	Radiomic features (9)	RP score ≥ 2	Logistic regression	0.750	SBRT for 14 stage I NSCLC patients
Cui et al.^14^	Dosimetric data (5), Clinical factors (13), Cytokines (30), miRNAs (62), SNPs (60)	RP grade ≥ 2	RF, SVM, MLP	0.831	CFRT for 106 NSCLC patients
Luna et al.^31^	Dosimetric data (11), Clinical factors (21)	RP grade ≥ 2	RF	0.66	CFRT for 203 stage II–III NSCLC patients

miRNAs, micro RNAs; SNPs, single nucleotide polymorphisms; RF, random forest; SVM, support vector machine; MLP, multilayer perceptron; CFRT, conventional fractionated radiotherapy.

RP grades were decided according to the Common Terminology Criteria for Adverse Events. RP scores were assigned based on identification of radiographic changes between pre- and post-RT CT images.

Previous studies used differences in radiomic features between pre- and post-treatment CT images for RP prediction^12,13^. However, this study predicted RP risk using only pretreatment planning CT images. Therefore, before new patients receive radiation therapy, it may be possible to determine the RP risk by applying treatment planning data to our RP predictive models. In addition, this method is reasonable in terms of clinical application as it requires only treatment planning data without additional clinical examinations.

This study has two limitations. First, only 22 (9%) of the 245 training patients included in this study had grade ≥ 2 RP. The imbalanced data was also a factor reducing the predictive model performance. To address these issues, the balanced subsets were sampled, and the ensemble averaging model was constructed using the 10 predictive models obtained from each subset. Second, we did not evaluate the repeatability and reproducibility of the radiomic features since we used only pretreatment planning CT. Traverso et al. reported that only radiomic features with high repeatability and reproducibility should be used in predictive models to reduce the risk of false-positive associations^32^. Therefore, to reduce the influence of radiomic feature variation on RP prediction as much as possible, we calculated the radiomic features under the same conditions for image acquisition settings, image reconstruction algorithm, digital image preprocessing, and software used to extract radiomic features in the training cohort. Moreover, the constructed models were tested in a separate test cohort, which was scanned on another equipment to validate repeatability and reproducibility. Nevertheless, these problems should be considered a limitation because texture features were less reproducible than histogram features^32^ and 11 of the 16 radiomic features selected as signatures for 10 subsets with four ROIs, shown in Table 3, were texture features.

In conclusion, the results of this study demonstrated the potential of RP predictive models after lung cancer SBRT using radiomic features for lung ROIs segmented by dosimetric information on pretreatment planning CT images. All radiomic models showed higher performance than the DVH model. The radiomic predictive model for LV5 was considered as the best model with a high AUC of 0.871 and 0.756 in both the training and test cohorts. Radiomic features calculated from pretreatment planning CT images can be used as imaging biomarkers for RP prediction in SBRT treatment planning for lung cancer.

## Supplementary information

Supplementary Figure S1.

Supplementary Table S1.

Supplementary Legends.
