# Supplementary figures and images for: Radiomic prediction of radiation pneumonitis on pretreatment planning computed tomography images prior to lung cancer stereotactic body radiation therapy
Source: Sci Rep. 2020 Nov 24;10:20424. doi: 10.1038/s41598-020-77552-7 (PMC7686358; doi:10.1038/s41598-020-77552-7)

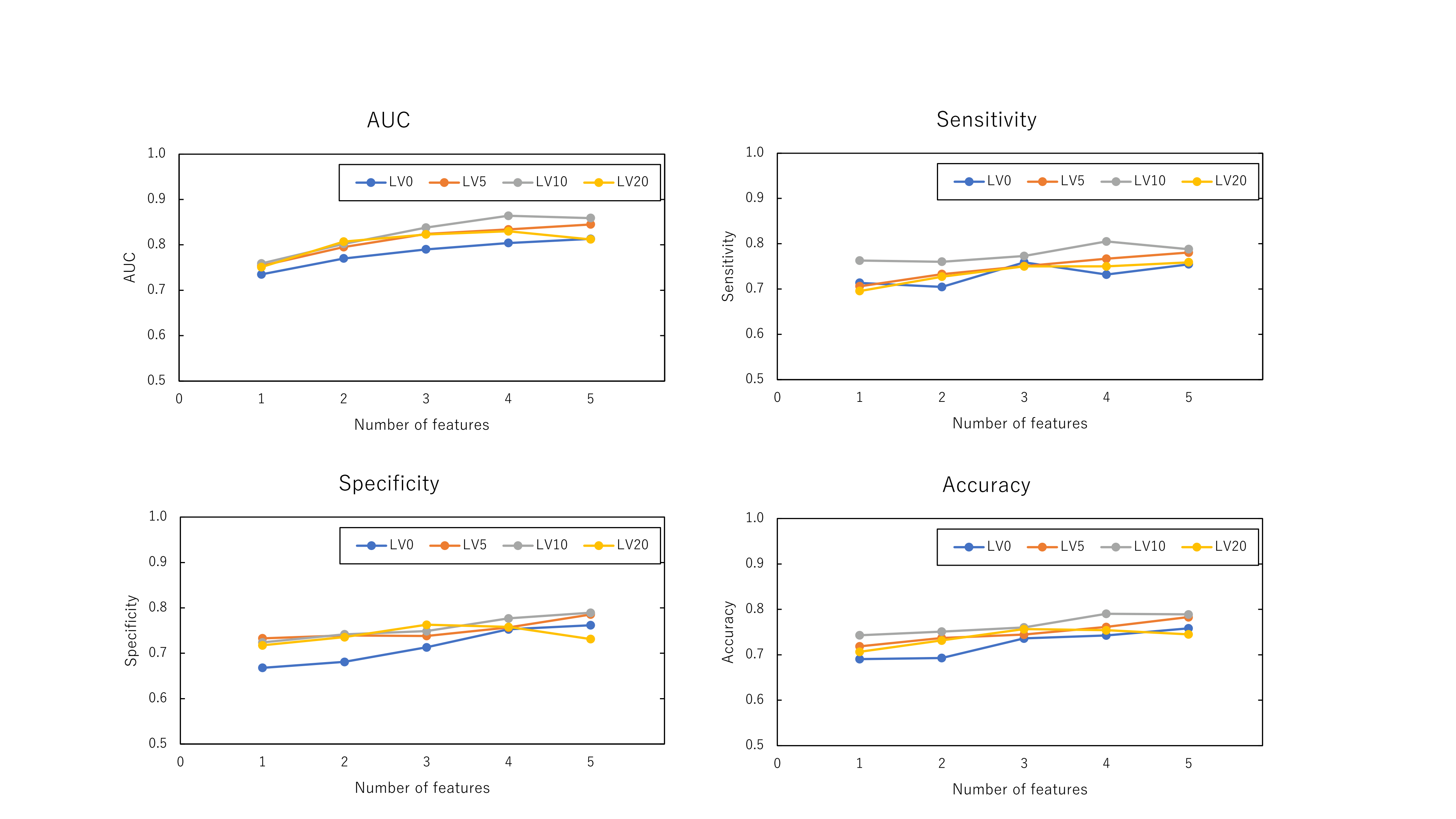

Supplement: Supplementary file 1 — Supplementary Figure S1. [file 41598_2020_77552_MOESM1_ESM.tif]
