# Supplementary material for: Radiomic prediction of radiation pneumonitis on pretreatment planning computed tomography images prior to lung cancer stereotactic body radiation therapy
Source: Sci Rep. 2020 Nov 24;10:20424. doi: 10.1038/s41598-020-77552-7 (PMC7686358; doi:10.1038/s41598-020-77552-7)
Supplement: Supplementary file 2 — Supplementary Table S1. [file 41598_2020_77552_MOESM2_ESM.doc]

**Radiomic prediction of radiation pneumonitis on pretreatment planning computed tomography images prior to lung cancer stereotactic body radiation therapy**

Taka-aki Hirose, Hidetaka Arimura, Kenta Ninomiya, Tadamasa Yoshitake, Jun-ichi Fukunaga, Yoshiyuki Shioyama

| **Table S1.** Radiomic features with the feature types. | | | | |
| --- | --- | --- | --- | --- |
| Histogram-based features (n = 14) | Texture features (n = 40) | | | |
|  | GLCM (n = 9) | GLRLM (n = 13) | GLSZM (n = 13) | NGTDM (n = 5) |
| Energy  Entropy  Kurtosis  Maximum  Mean  Mean absolute difference  Median  Minimum  Range  Root mean square  Skewness  Standard deviation (SD)  Uniformity  Variance | Energy  Contrast  Entropy_GLCM  Homogeneity  Correlation  Variance  Sum average  Dissimilarity  Auto correlation | Short run emphasis (SRE)  Long run emphasis (LRE)  Gray level non-uniformity (GLN)  Run length non-uniformity (RLN)  Run percentage (RP)  Low gray level run emphasis (LGRE)  High gray level run emphasis (HGRE)  Short run low gray level emphasis (SRLGE)  Short run high gray level emphasis (SRHGE)  Long run low gray level emphasis (LRLGE)  Long run high gray level emphasis (LRHGE)  Gray level variance (GLV)  Run-length variance (RLV) | Small zone emphasis (SZE)  Large zone emphasis (LZE)  Gray level non-uniformity (GLN)  Zone-size non-uniformity (ZSN)  Zone percentage (ZP)  Low gray level zone emphasis (LGZE)  High gray level zone emphasis (HGZE)  Small zone low gray level emphasis (SZLGE)  Small zone high gray level emphasis (SZHGE)  Large zone low gray level emphasis (LZLGE)  Large zone high gray level emphasis (LZHGE)  Gray level variance (GLV)  Zone-size variance (ZSV) | Coarseness  Contrast  Busyness  Complexity  Strength |

GLCM: Gray level Co-occurrence Matrix; GLRLM: Gray level Run-Length Matrix; GLSZM: Gray level Size Zone Matrix; NGTDM: Neighborhood Gray-Tone Difference Matrix
