# Supplementary material for: Radiomic prediction of radiation pneumonitis on pretreatment planning computed tomography images prior to lung cancer stereotactic body radiation therapy
Source: Sci Rep. 2020 Nov 24;10:20424. doi: 10.1038/s41598-020-77552-7 (PMC7686358; doi:10.1038/s41598-020-77552-7)
Supplement: Supplementary file 3 — Supplementary Legends. [file 41598_2020_77552_MOESM3_ESM.docx]

Figure S1. The mean AUCs, sensitivity, specificity, and accuracy of 10 subsets with increasing number of top five features for RP prediction with each ROI.

Table S1. Radiomic features with the feature types.
